# Impact of Common Food Labels on Consumer Liking in Vanilla Yogurt

**DOI:** 10.3390/foods8110584

**Published:** 2019-11-17

**Authors:** Theresa Li, Robin Dando

**Affiliations:** Department of Food Science, Cornell University, Ithaca, NY 14853, USA; tl736@cornell.edu

**Keywords:** taste, labels, sensory analysis, yogurt, extrinsic cues

## Abstract

As competition on super market shelves is higher than ever, the importance of product concepts, communicated through labels, can dictate a product’s success or failure. However, it is possible for labels to affect a consumer’s experience, changing the overall response to the product. In this study, we tested samples of vanilla yogurt with one of four commonly used labeling concepts (high-protein, low-fat, made with stevia and all-natural) on sensory perception, consumer liking, expected consumption amount, and willingness to pay (WTP) in a consumer test (*n* = 108). Each participant evaluated five samples of the same vanilla yogurt identified with one of the labels, or an unlabeled control. Results showed panelists liked the samples labeled with low-fat and high-protein to the greatest degree, with all-natural scoring the lowest. Those more concerned with protein content found the samples less satiating, dependent on sex. Sweetness was also perceived more highly in younger panelists, with panelists WTP dependent on their liking of the labels. Results highlight the importance of labeling as an extrinsic cue affecting liking ratings, with potential ramification for ultimate product success. Understanding consumers’ response to labels, as well as their attitudes, has broad implications for food marketing, as well as public health and the study of eating habits.

## 1. Introduction

As competition in the processed food industry remains heated, companies continue to utilize extrinsic cues to establish product expectations and attract consumers. Labels can establish an expectation of a product’s sensory or hedonic properties long before consumption, which may be reinforced or contradicted when actually tasting the product. Expectation in foods is a belief about a product [1] existing before consumers experience the physical product itself [2]. Extrinsic cues are pieces of information directly related to this product, though not contained within the actual product itself; they are usually the first encounter consumers have with a food. Expectations can be powerful, as consumers tend to shift their perception toward a direction of perceived expectation (assimilation) when a discrepancy in outcome occurs [3]. Some of the most common extrinsic cues to set up expectations around a food are from food labels that can convey a variety of information about a food product, including sensory descriptions, processing steps, ethical values, and nutrition-related information.

Many studies have shown how labels affect consumer expectation, and ultimately, consumption behavior [4,5]. In previous work about the effect of healthy ingredients, growth origin, and process labels (“local”, “organic”, “free-range”, etc.), sensory and nutritional evaluations were more likely to be positive with such an expectation established [6,7], possibly due to some form of positive Halo effect (influencing unrelated areas due to an impression from another area [8]) from these positively regarded terms. However, another study from De Pelsmaeker et al. [9] using conjoint analysis suggested that taste may still be the key factor defining consumer preference. The current study focused on the effects of labels alone, without changing the product itself, with labels identical in image, font, color, and design, aside from wording. For the current study, “all-natural”, “low-fat”, “high-protein”, and “made with Stevia” were selected as commonly used modern claims, with vanilla yogurt selected as a product well suiting these 4 claims.

“All-natural” was selected not only because of its current prevalence in the marketplace but also as the term is not yet clearly defined or regulated [10]. “Natural” labels may evoke an idea of nature, and thus positive feeling toward the food and its relation to health benefits [11]. Participants perceive all-natural products to have better taste/quality, nutrition [12,13,14], as well as safer processing methods [15]. Past research also found a higher Willingness to Pay (WTP) associated with “natural” labeled foods [16].

The demand for “high-protein” products has been increasing over the past decade. 55% of US households consider “high-protein” an important attribute when they shop for food [17]. High-protein samples are often judged as more satisfying compared to lower-protein versions [18]. Yogurt with 24 g of protein was also found to be more effective in reducing hunger, increasing fullness, and delaying subsequent eating, compared to lower-protein yogurt [19].

One of the most prevalent claims in food labeling is low-fat. Despite changing opinions on fat [20], it is often associated with higher risks of cardiovascular disease, and a high caloric content [21]. Low-fat alternative foods are commonly recommended for weight management [22]. Participants sampling milkshakes with an expectation for low-fat products reported higher liking than those who expected high-fat milkshakes [23]. However, conversely, Norton et al. [24] found low-fat labeling can have an adverse effect on expected liking; however, no effect on actual liking or rating of sensory attributes was reported. Kähköne et al. [25] also found that low-fat claims had no effect on pleasantness or sensory rating of strawberry yogurt.

Low-sugar is a common demand for processed foods [26]. Thus, natural noncaloric sweeteners such as Stevia have become very popular. Younger consumers favored formulations containing stevia versus older generations [27]; however, the influence of an expectation for stevia is not clear, with some studies showing preference for stevia labeled samples from label conscious parents [28] and others showing a negative emotional conceptualization when sugar is replaced by stevia [29].

In a 1993 conjoint analysis of taste [30], health claims, price, and brand for purchasing strawberry yogurt showed taste and health claims had the most significant influence on buying intent. However, in over 25 years since this study, a number of new trends have emerged, whose influence on expectations remain unclear. Additionally, previous work has largely neglected the background of panelists; it is also unknown if a panelist’s demographic group, or perception of health claims influences such responses. The current study was designed to address such gaps. Our objectives were 1) to investigate the influence of common food labels on consumer liking if only labels were changed; and 2) to further evaluate the effect of labels on perceived consumption levels, and willingness to pay. We used yogurt as a model for the current study due to well-documented nutritional benefits [31], its established sensory baseline [32], and its ability to convey all four labels of interest. It is also common for yogurt to be flavored and modified by sweeteners [33]. In the U.S., even though dairy consumption is promoted through nutrition education, yogurt consumption is relatively low. According to United States Department of Agriculture (USDA), the per capita consumption for yogurt in 2018 is 13.4 pounds per person compared to 146 pounds per person for milk [34]. According to a Luckhow et al. [35], U.S. yogurt consumers are divided into segments, some devoted to health, and others to sensory experience. Research has also found that yogurt consumption is associated with better diet quality and metabolic profile in Americans [36], with consumers having higher diet quality and healthier life styles compared to non-consumers [37].

## 2. Material and Methods

All study procedures were approved by the Institutional Review Board for Human Participants at Cornell University (Ithaca, NY, USA). A total of 108 panelists were recruited from the local community, through poster advertisement and an email listserv provided by the Cornell Sensory Evaluation Center. All participants were prescreened as yogurt consumers, and to exclude any allergies or intolerance to dairy products, with informed consent given by all participants. The participants were told the study was a commercial sensory evaluation for a small dairy company, but were not aware of the full purpose of the research.

Each session took 20–30 min. The participants tested samples in individual booths in the Sensory Evaluation Center at Cornell University, using RedJade sensory evaluation software (RedJade Sensory Solutions LLC, USA) to collect sensory ratings, under red lights, to disguise visual cues and aid in hiding the study’s true purpose. The samples were delivered in a counterbalanced order, each with a three-digit code, and kept at 4 degrees Celsius before serving directly from the refrigerator. All participants were briefly trained [38] on the generalized Labeled Magnitude Scale (gLMS) before they started the evaluation, using standard scale points of no sensation (0) barely detectable (1.4), weak (6), moderate (17), strong (35), very strong (51), strongest imaginable sensation of any kind (100). The panelists were required to inspect the label for 60 seconds and rate their liking of it before tasting or answering any questions about the sample, which also acted as an enforced pause between samples, when they were also prompted to cleanse their palate with water and crackers. Each sample was then tasted and evaluated for overall liking on the Labeled Affected Magnitude (LAM ) scale [39] with anchors at 0 (greatest imaginable dislike), 16 (dislike extremely), 27 (dislike very much), 40 (dislike moderately), 47 (dislike slightly), 50 (neither like nor dislike), 53 (like slightly), 70 (like moderately), 73 (like very much), 83 (like extremely), and 100 (greatest imaginable like), as well as for thickness, creaminess, and sweetness on the gLMS. Panelists were also asked to describe an aftertaste if they detected one. Participants rated their willingness to pay for a single serving (WTP, ranging from $0 to $4) and how much they thought they could consume (on a scale of 0 oz to 12 oz with panelists advised a usual yogurt serving is 4 oz) in one occasion, to test predicted satiating properties of the described sample. Panelists also rated their liking of each label from 0 to 100 on the LAM scale, and rated the importance of low sugar, low fat, high protein, and all-natural claims to their purchasing habits on a 100-point unstructured line scale, then answered a series of demographic questions at the end of the study.

**Figure 1:**
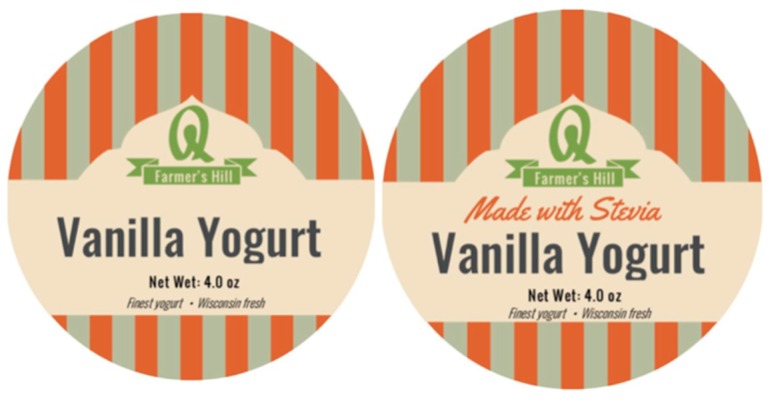
Labels used to identify samples denoting no claims (control) and label showing “Made with Stevia” claim.

The vanilla yogurt used in this study was obtained from the Cornell Dairy (Ithaca, NY, USA) and was divided into 1 oz samples in transparent plastic containers in the sensory kitchen. The labels were designed with the purpose of disguising the study as a commercial sensory evaluation (see Figure 1), with labels matching in color, size, font, and graphics, with only the description differing. In total, 5 samples were served, including one sample without a claim on the label (termed unlabeled). Although taste may be key in determining liking [9], to isolate the effects of labeling, all products tested were identical.

In the initial analysis, a repeated measure one-way analysis of variance (ANOVA) was performed with Prism 5.0 (GraphPad Software, San Diego, CA, USA) to investigate the effects of labels on each dependent variable. In a more detailed analysis, a linear mixed model was built using SPSS 24 from IBM (Armonk, NY, USA). Tests yielding *p* values below 0.05 were assumed to be statistically significant.

## 3. Results

**Figure 2:**
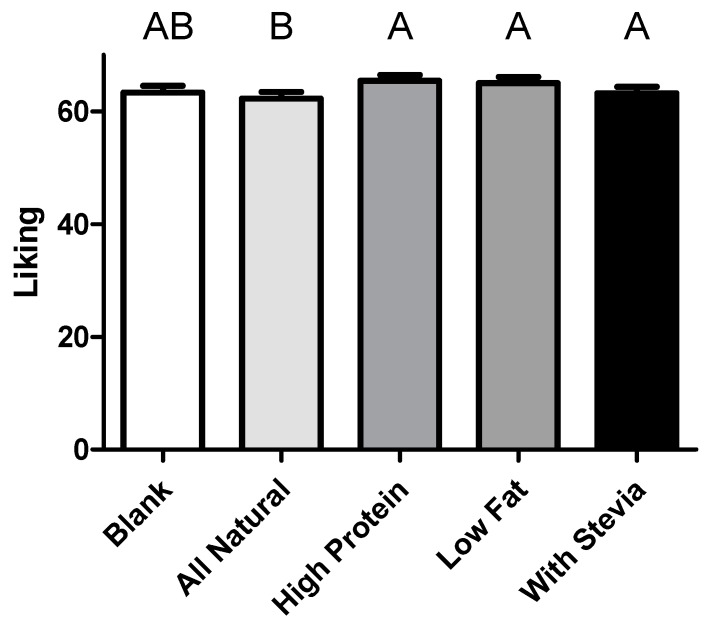
Overall liking of samples rated from 0 to 100 with anchors at 0 (Most imaginable disliking), 16 (Dislike extremely), 27 (Dislike very much), 40 (Dislike moderately), 47 (Dislike slightly), 50 (Neither like nor dislike), 53 (Like slightly), 70 (Like moderately), 73 (Like very much), 83 (Like extremely), and 100 (Most imaginable liking). Letters denote significant differences. Bars denote mean plus standard error.

108 panelists completed all parts of the test (82 female), with all identifying as yogurt consumers, and 103 eating yogurt at least once a week. 46% of the panelists were between age 18 and 25 with a further 28% between 26–35, and all others above 36 years old. Panelists most highly rated the importance of low sugar (median = 40.5 on 100 pt scale) and high protein (median = 38.5) claims guiding their buying habits; however, low-fat and all-natural were not far behind (median = 30 for both). The labels displayed influenced a panelist’s overall liking of the sample (Figure 2, *p* = 0.042), with high protein and low fat labeled samples liked the most, and all-natural liked less than the other claims.

**Figure 3:**
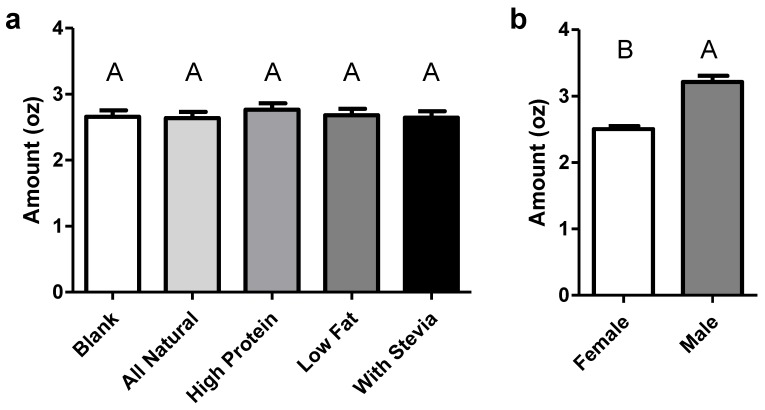
Ideal amount of consumption by label (**a**) and sex (**b**). Y-axis denotes ounces, with panelists advised a usual serving is 4 oz. Letters denote significant differences. Bars denote mean plus standard error.

Panelist’s age and sex also affected overall liking of the samples, with younger panelists liking samples more than older (*p* = 0.012) and females liking samples more than males (*p* = 0.017). Those identifying low sugar (*p* = 0.020) and low fat (*p* = 0.002) claims as important also rated samples as higher in liking across the board. Finally, the degree to which a panelist liked the label affected their overall liking of the samples (*p* < 0.001). No strong trends were appreciated in panelists’ perception of thickness, creaminess, or sweetness in the samples from the labels or from any other factors tested, aside from a higher reported level of perceived sweetness in younger panelists. In rating their ideal serving size for the samples, panelists’ liking for the label was a significant factor in the analysis (*p* < 0.001), however, the label itself was not (Figure 3), unlike panelist sex (*p* < 0.001), with males wanting to consume significantly more than females, and the importance of protein claims, with those finding them important again wanting to consume larger portions (*p* = 0.019).

**Figure 4:**
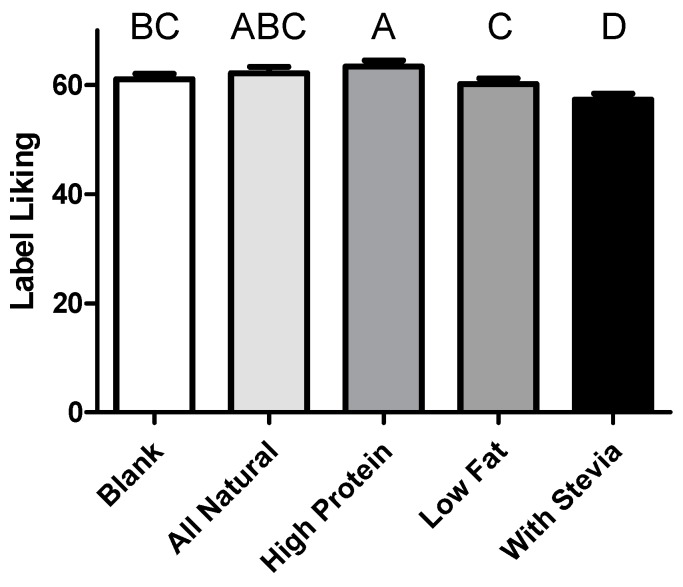
Panelist’s rated their liking of each label rated from 1 to 100 with anchors at 0 (Most imaginable disliking), 16 (Dislike extremely), 27 (Dislike very much), 40 (Dislike moderately), 47 (Dislike slightly), 50 (Neither like nor dislike), 53 (Like slightly), 70 (Like moderately), 73 (Like very much), 83 (Like extremely), and 100 (Most imaginable liking). Letters denote significant differences. Bars denote mean plus standard error.

Willingness to pay was dictated only by a panelist’s liking for the label attached to the samples (*p* < 0.001), although samples’ WTP was somewhat a reflection of liking scores, with high protein highest, followed by low-fat, similar to liking. Despite similar ordering, label did not statistically affect WTP (*p* = 0.161). The appeal of the labels differed significantly (*p* < 0.001), despite only wording changing between presentations. The most appealing labels were “high-protein” and “all-natural” respectively (Figure 4), with “made with stevia” the least appealing and “all-natural” only slightly better, with both liked less than those with no identifying claims at all.

## 4. Discussion

The goal of the study was to analyze the influence of popular labels (all-natural, low sugar, low fat, and made with stevia) on the liking of yogurt. Results demonstrated higher liking for samples labeled as high-protein and low-fat, with an all-natural claim leading to the lowest level of liking. Our findings support those of previous market research suggesting that consumers prefer high protein in yogurt samples, as well as work showing low-fat samples were rated as better tasting [17,23,40,41]. This consistency supports the importance of labels in determining food preference, highlighting potential implication in food marketing and consumer behavior. These findings may also have implications for regulatory agencies such as the Food and Drug Administration (FDA), to further refine their labeling guidelines. In previous studies, improved taste was reported for yogurt labeled as all-natural [12], despite its lack of a firm legal definition, with no effect on sensory ratings for yogurt labelled as low-fat [25]. Our results are some of the first to detail the effect of high-protein and made-with-stevia labeling on consumer responses.

The study also showed that the ideal amount to be consumed on one occasion was significantly higher in males than females. This is supported by both physiological and psychological research. Males generally need more energy than women. According to the 2015–2020 Dietary Guidelines, the estimated daily caloric needs for males 18 years old is 2400 kcal, where it is 2000 kcal for females of the same age [42]. Past research also suggests that women are more likely than men to be dieting due to health reasons or dissatisfaction with their weight [43,44,45]. In the search for ever-greater segmentation among products, this work may highlight a potential for differing serving sizes of yogurt, targeted at sex-based segments, in the future.

Few differences were perceived in the sensory notes arising from the yogurt samples by label. This may be to be expected, as features on the label were purposefully kept identical between the samples (shape [46], color [47,48], and font [49] have all been demonstrated to impose forms of crossmodal influence on flavor perception); however, this further highlights the importance of expectation set up by a label, whereby samples were sensorially equivalent, but nonetheless one was still liked more than another, presumably due to an expectation set up by the labeling. There was also a significant effect of a specific label on liking of the samples, affecting liking scores more strongly than the identity of the labels alone. Sweetness of the samples was perceived differently depending on age; younger panelists considered the same samples to be sweeter than older panelists. This result matches those of a number of studies that suggest that older subjects are less sensitive to taste stimuli [50,51,52], as well as that age is a significant factor in determining desired levels of sweetness [53].

Unlike many studies that found high-protein foods are more satiating [19,54], results suggested that merely labeling a product “high-protein” was not sufficient to induce a higher level of satiety, at least extrapolated from desired portion sizes as reported here. However, those seeing importance in high-protein claims did desire larger portion sizes. WTP associated with the liking of the labels, implying that willingness to pay depended on how a claim appealed to the consumer, rather than simply the identity of the claim itself, highlighting the importance of segmentation in yogurt marketing. There are a number of studies indicating higher WTP for natural and organic products, while suggesting some demographic factors such as gender and age affected WTP [55,56,57]; however, these factors did not display a significant impact in our test, possibly due to the demographics of our panelists.

In the current study, we failed to see any preference for natural claims or an “attitude-liking” gap as addressed in Hemmerling’s work [58]. The level of education of the panelists might be a factor accounting for this difference, with our demographics representing a college population, and further one primarily drawn from a Food Science department. Low-fat labeling has also been reported to have the potential for an adverse effect on expected liking [24]. Interestingly, panelists found the “made with stevia” label to be the least appealing, in line with the idea that ] not all consumers find stevia to be an acceptable additive in foods [59], likely dependent on education [60]. Nonetheless, links between healthy eating, diet, and sensory function are significant in populations such as the one within this study [53].

The implications from our study regarding the labeling of products are that a product’s hedonic response can be influenced by simply identifying the product in a differing manner, regardless of sensory properties. This effect seems strongly determined by an individual’s liking of a particular label or concept. Since our study was performed in a university sensory center, the demographics of the panelists may be considered one limitation, failing to match the demographics of the US population regarding age, gender balance, or ethnicity. The study was primarily female participants (76%), with many people 18–25 years old (46%). This may at least partially explain why responses to labels differed in part from those previously published, as trends and demographics shift with time. The sample size of 108 panelists may also be considered small by some. Furthermore, the study tested a single product (vanilla yogurt), which is generally considered as a relatively healthy food. It is unclear how results would vary when similar labels were applied to less healthy foods, for example, potato chips or other snack foods. Lastly, each panelist tasted the same samples in each presentation, with only labels varying. In order to attempt to hide the study’s true purpose, samples were assessed under red lights, to disorient panelists slightly, with mandatory breaks enforced between samples to reduce the likelihood of a panelist realizing the samples were exactly the same as the previous sample. Thus, the inherently superior control of a within-subjects design may have been somewhat counteracted by the possibility that some suspected the yogurts were from the same source, despite the claims on the labels. Nonetheless, differences in overall liking, willingness to pay, and a panelist’s ideal consumption level with label, or liking of label, highlights the importance of extrinsic cues such as those found on labels on a product’s performance in the market.
